# Oxidative Stress Triggers Defective Autophagy in Endothelial Cells: Role in Atherothrombosis Development

**DOI:** 10.3390/antiox10030387

**Published:** 2021-03-05

**Authors:** Cristina Carresi, Rocco Mollace, Roberta Macrì, Miriam Scicchitano, Francesca Bosco, Federica Scarano, Anna Rita Coppoletta, Lorenza Guarnieri, Stefano Ruga, Maria Caterina Zito, Saverio Nucera, Micaela Gliozzi, Vincenzo Musolino, Jessica Maiuolo, Ernesto Palma, Vincenzo Mollace

**Affiliations:** 1 Research for Food Safety & Health IRC-FSH, Department of Health Sciences, University Magna Graecia, 88100 Catanzaro, Italy; rocco.mollace@gmail.com (R.M.); robertamacri85@gmail.com (R.M.); miriam.scicchitano@hotmail.it (M.S.); boscofrancesca.bf@libero.it (F.B.); federicascar87@gmail.com (F.S.); annarita.coppoletta@libero.it (A.R.C.); lorenzacz808@gmail.com (L.G.); rugast1@gmail.com (S.R.); mariacaterina.zito@libero.it (M.C.Z.); saverio.nucera@hotmail.it (S.N.); gliozzi@unicz.it (M.G.); v.musolino@unicz.it (V.M.); jessicamaiuolo@virgilio.it (J.M.); palma@unicz.it (E.P.); mollace@libero.it (V.M.); 2 Nutramed S.c.a.r.l., Complesso Ninì Barbieri, Roccelletta di Borgia, 88100 Catanzaro, Italy

**Keywords:** oxidative stress, autophagy, endothelium, atherosclerosis, atherothrombosis

## Abstract

Atherothrombosis, a multifactorial and multistep artery disorder, represents one of the main causes of morbidity and mortality worldwide. The development and progression of atherothrombosis is closely associated with age, gender and a complex relationship between unhealthy lifestyle habits and several genetic risk factors. The imbalance between oxidative stress and antioxidant defenses is the main biological event leading to the development of a pro-oxidant phenotype, triggering cellular and molecular mechanisms associated with the atherothrombotic process. The pathogenesis of atherosclerosis and its late thrombotic complications involve multiple cellular events such as inflammation, endothelial dysfunction, proliferation of vascular smooth muscle cells (SMCs), extracellular matrix (ECM) alterations, and platelet activation, contributing to chronic pathological remodeling of the vascular wall, atheromatous plague formation, vascular stenosis, and eventually, thrombus growth and propagation. Emerging studies suggest that clotting activation and endothelial cell (EC) dysfunction play key roles in the pathogenesis of atherothrombosis. Furthermore, a growing body of evidence indicates that defective autophagy is closely linked to the overproduction of reactive oxygen species (ROS) which, in turn, are involved in the development and progression of atherosclerotic disease. This topic represents a large field of study aimed at identifying new potential therapeutic targets. In this review, we focus on the major role played by the autophagic pathway induced by oxidative stress in the modulation of EC dysfunction as a background to understand its potential role in the development of atherothrombosis.

## 1. Introduction

Atherosclerosis is a chronic cardiovascular condition that occurs in the arterial and venous circulations, representing a major risk factor that predisposes sufferers to several cardiovascular diseases (CVDs) such as myocardial infarction (MI), ischemic stroke, and thromboembolism [1]. Currently, CVDs are the main cause of morbidity and mortality worldwide, and their incidence is greater in elderly people and in patients with diabetes, metabolic disorders, and hypertension, who are particularly vulnerable to the development of atherosclerosis and related thrombotic complications. The increase in life expectancy, which is estimated to further increase in the next decades, highlights the clinical and socioeconomic impacts of CVD management [2]. Atherosclerosis is a progressive inflammatory process in which lipid accumulation represents the key factor in the early phase of the disease. In pathological conditions, atherogenic lipoproteins, such as low-density lipoproteins (LDLs), spread and accumulate in the subendothelial space of the arterial wall where they undergo progressive oxidative modifications to form oxidized LDLs (oxLDLs) [3]. LDLs are highly susceptible to modification by the oxidative milieu found inside the vascular wall, and their retention in predisposed areas induces an inflammatory response characterized by the increased expression of chemotactic proteins, such as monocyte chemoattractant protein-1 (MCP-1), vascular cell adhesion molecule-1 (VCAM-1), and P and E- selectins on the endothelial cells (ECs) [4,5]. Enhanced expression of cell adhesion molecules leads to the recruitment and infiltration of leukocytes into the subendothelial space and up to the intimal layer. Then, monocytes differentiate into macrophages and engulf oxLDLs, becoming foam cells and contributing to plaque/atheroma development by secreting multiple mediators of the inflammatory process in the vessel wall [6]. The recruitment of circulating monocytes and T-cells due to the increased inflammation triggers the migration of vascular smooth muscle cells (SMCs) from the tunica media into the subendothelial space where they abnormally proliferate and secrete extracellular matrix (ECM) proteins, contributing to atheroma growth [7]. The latter are mainly composed of a mixture of macrophages, lymphocytes, a few SMCs, cholesterol cleft, necrotic debris, and lipid-laden foam cells and, in the advanced stages, are also characterized by intra-plaque neovascularization, hemorrhages, and a thin fibrous cap. Platelet aggregation and clotting activation play pivotal roles in the development of thrombotic complications by adhering to the exposed subendothelium at the site of plaque rupture and erosion [8]. The rupture of the fibrous caps of such vulnerable plaques also leads to luminal thrombosis, arterial occlusion, or embolism in areas of the vascular bed far from the atherosclerotic area [9]. 

Emerging evidence suggests that together with clotting system activation, the dysfunction of the EC layer represents a main mechanism implicated in the occurrence and progression of atherothrombosis, as it is a common process associated with both artery and venous thrombosis [10]. Indeed, the exacerbation of oxidative stress is closely involved in the remodeling of the vessel wall, resulting in nitric oxide (NO)-related endothelial dysfunction. The subsequent imbalance between vasoconstriction and vasodilatation increases the endothelial permeability and triggers a local inflammatory response [4]. Reactive oxygen species (ROS) are considered crucial mediators of vascular homeostasis and the atherosclerosis pathogenesis [11] 

Under physiological conditions, ROS and reactive nitrogen species (RNS), released in moderate concentrations, are essential for the regulation of multiple cellular processes such as cell signaling, cell cycle regulation, apoptosis, and gene expression through interactions with transcription factors [12]. However, an imbalance between the concentration of ROS/RNS and the endogenous antioxidant capacity exacerbates the oxidative stress process [13]. ROS especially promote the oxidative modification of LDL but also modify lipids, proteins, and DNA, leading to the progression of disease [14]. The main direct or indirect sources of ROS related to the onset of atherothrombosis are closely linked to the activation of pro-oxidant pathways such as NADPH oxidase (NOX), myeloperoxidase (MPO), uncoupled endothelial nitric oxide synthase (eNOS), and lipoxygenases [2,15]. 

In recent years, experimental evidence has identified ROS/RNS as early inducers of autophagy upon nutrient deprivation, although the molecular mechanism driving this process remains to be fully elucidated [16,17]. Autophagy is an evolutionary conserved catabolic pathway for bulk degradation that plays a key role in eliminating long-lived proteins, macromolecular aggregates, and damaged intracellular organelles and simultaneously generates an internal nutrient pool of macromolecules needed to support metabolic reactions [18,19]. These processes are essential both to maintain cellular homeostasis and to provide a survival mechanism in response to various stressful conditions, such as nutrient deprivation, viral infections, and genotoxic stress [20]. A number of autophagy-related proteins (Atgs) organized as functional complexes are essential for the formation of the autophagosome and its subsequent fusion with pre-existing lysosomes to degrade and recycle their cargo [21]. Vital processes, such as cellular growth and proliferation, are regulated through autophagy by modulating the energy demand with the extracellular stimuli and amino acids and glucose availability [20]. Thus, it is generally assumed that autophagy represents a pro-survival mechanism, although its deregulation has been linked to non-apoptotic cell death. 

Recent findings highlight the role of autophagic flux in maintaining normal vessel wall biology and therefore playing a pivotal role in EC homeostasis [22]. Moreover, several studies have shown a clear link between autophagy and a wide array of vascular processes, ranging from angiogenesis to calcification of the vessel wall, as well as in the paracrine regulation of vasoactive substances from the endothelium [23,24]. According to this evidence, autophagic dysregulation may also be implicated in vascular pathological remodeling and disease processes such as atherosclerosis, leading to EC dysfunction and contributing to the development of inflammation and thrombosis [25,26].

Thus, there is a growing interest in understanding the contribution of autophagy to vascular homeostasis and the onset and development of atherosclerotic disease with the aim of identifying some specific process targets that could be used for future therapeutic benefit.

In this review, we briefly summarize the role of oxidative stress in atherothrombosis and focus on the link between oxidative stress and defective autophagy in ECs during the atherothrombotic process.

## 2. Role of Oxidative Stress in Vascular Haemostasis

ROS are important intermediates that function as secondary messengers within cells. An imbalance between ROS production and cellular antioxidant system activity is the main cause of endothelial dysfunction induction, leading to vascular injury in several metabolic disorders and, particularly, atherosclerosis [27]. Indeed, chronic inflammation and the overproduction of free radicals is the main mechanism capable of enhancing hemostasis through the development of platelet hyper-reactivity, which is associated with a significant thrombotic risk [28,29,30]. Recent studies showed that platelets could be a target of ROS but also a source of free radicals. Indeed, an imbalance between ROS production and the antioxidant system dysregulates and amplifies platelet activation due to isoprostane formation, the modulation of platelet receptors, and the oxidation of LDLs. Platelet activation, in turn, leads to further ROS production through NOX activation, triggering mitochondrial dysfunction [31,32]. The hemostatic process represents a complex cascade that can be distinguished into three subfractions: the first step consists of primary hemostasis, in which an interaction between platelets and the damaged endothelium occurs; the second phase is secondary hemostasis carried out through the activation of the clotting system, resulting in the production of fibrin clots; and the last step is fibrinolysis [33].

### 2.1. Primary Haemostasis and Platelet Activation 

**Figure 1:**
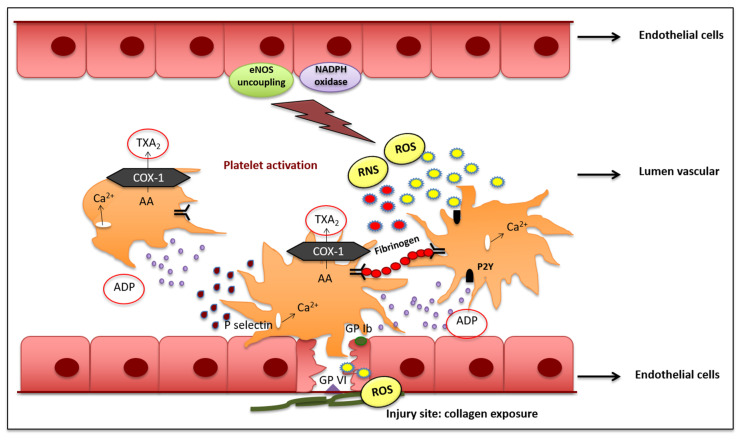
Platelet activation following exposure to collagen at the injury site and the production of free radicals. Protein tyrosine-phosphatases (PTPs) are the primary targets of ROS which, in turn, drive the upregulation of GPVI signaling. This event results in the overstimulation of platelets, inducing cytosolic calcium mobilization and the release of proaggregating factors from granules. Due to endothelial dysfunction, uncoupled eNOS uncoupling and NOX activation lead to the overproduction of ROS and RNS which are able to interact with platelets through the ADP receptor, triggering the same intracellular signaling pathway. Furthermore, nitrogen peroxide triggers the activation of fibrinogen and the stabilization of the fibrin clot. Thus, endothelial dysfunction and platelet activation in primary hemostasis, both consequences of a pro-oxidant state, converge and amplify each other, causing important prothrombotic effects. AA: Arachidonic Acid; COX-1: Cyclooxygenase-1; GP Ib: glycoprotein Ib; GPVI: glycoprotein VI; eNOS: endothelial Nitric Oxide Synthase; NOX: NADPH oxidase; P2Y: Peptide 2Y; RNS: Reactive Nitrogen Species; ROS: Reactive Oxygen Species; TXA2: Thromboxane A2.

Primary hemostasis is a platelet activation process that is carried out through the recruitment of platelets to the site of vascular injury and involves the adhesion, activation, and recruitment of platelets [34]. In the platelet adhesion phase, there is a deceleration of platelets mediated by the von Willebrand factor (vWF), which binds to the collagen fibers of the exposed endothelium at the site of injury, forming a connection between the collagen matrix and the platelet glycoprotein Ib-IX-V complex. The activated platelet monolayer releases proinflammatory and proaggregating factors from their granules [8]. This process triggers further recruitment of platelets into the vessel site of injury from the circulation, leading to the formation of platelet aggregates. Activated platelets release adenosine diphosphate (ADP) and thromboxane A2 (TXA2), inducing the production of secondary messengers, such as diacylglycerol (DAG) and inositol 1,4,5-trisphosphate (IP3) which, in turn, lead to an increased concentration of cytosolic calcium [35,36]. Del Principe et al. conducted one of the first studies to highlight the key role of free radicals and, in particular, of hydrogen peroxide (H_2_O_2_), in platelet activation induced by collagen through significant calcium mobilization [37]. These data were confirmed in recent studies, proving that the primary targets of free radicals produced by collagen activation are the protein tyrosine-phosphatases (PTPs), which are able to upregulate glycoprotein VI (GPVI) signaling. Indeed, tyrosine phosphorylation is controlled by the balanced actions of protein-tyrosine kinases (PTKs) and PTPs; in particular, oxidation of the catalytic cysteine of PTPs leads to their inactivation, with consequent uncontrolled phosphorylation and upregulation of several platelet collagen receptors, including GPVI. The uncontrolled activation of GPVI induces platelet hyperstimulation, thereby enhancing cytosolic calcium mobilization, the release of proaggregating factors from granules, and consequent thrombus formation [38,39] (Figure 1). Further experimental studies showed that ROS are able to mimic the roles of ADP and IP3 by influencing primary hemostasis through platelet activation [40].

### 2.2. Secondary Haemostasis and Clotting Formation 

**Figure 2:**
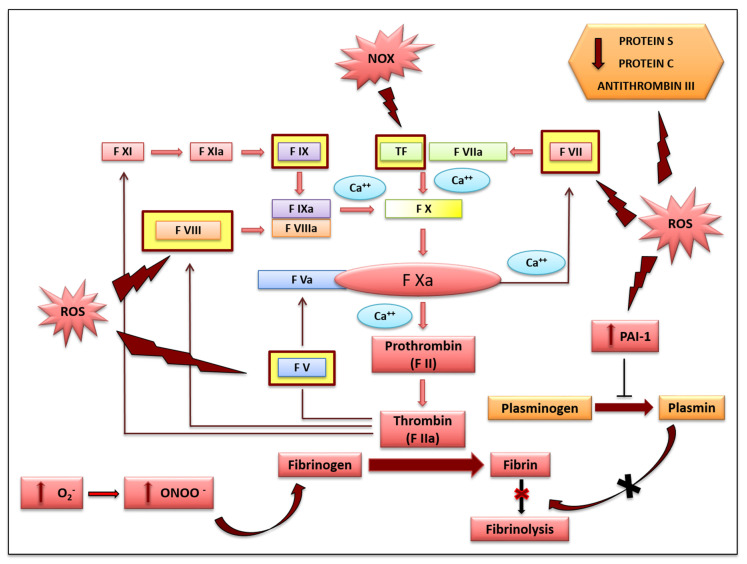
The figure shows ROS and NOX modulation of different phases of secondary hemostasis and fibrinolysis through the upregulation of several proteases and the inhibition of antithrombotic factors in ECs. The imbalance generated between prothrombotic and antithrombotic factors leads to the stabilization of fibrin clot formation with subsequent development of the atherothrombotic process. ECs: Endothelial Cells; F V; Factor V; F Va; Factor Va; F VII; Factor VII; F VIIa: Factor VIIa; F VIII: Factor VIII; F VIIIa: Factor VIIIa; F IX: Factor IX; F IXa: Factor IXa; F X: Factor X; F Xa: Factor Xa; F XI: Factor XI; F XIa: Factor XIa; PAI-1: Plasminogen Activator Inhibitor-1; ROS: Reactive Oxygen Species; NOX: NADPH oxidase; TF: Tissue Factor.

The clotting system plays a key role in the development of thrombosis and consists of activation of the protease cascade, which results in fibrin formation. This mechanism is an important defense process following vessel injury, but its dysregulation leads to thrombus formation [41,42]. Increasing evidence clearly shows the impact of clotting system dysfunction on the hemostasis process, highlighting the determinant effect of ROS overproduction in several in vitro and in vivo models [43]. Increase of different proteases involved in the coagulation cascade, such as procallicrein (Factor V), proconvertin (Factor VII), antihemophilic A factor (Factor VIII), and antihemophilic B factor (Factor IX), exaggerate the activation of the Stuart factor (Factor X) which, once activated, leads to the generation of thrombin from prothrombin and, therefore, fibrin from fibrinogen. This impairment leads to disproportionate clotting formation. In particular, it has been observed that ROS, especially those generated by NOX, promote clotting activation, as they are able to upregulate the tissue factor (Factor III) in leucocytes and ECs [44,45]. Indeed, the alteration of ECs is the main cause of prothrombotic effects within blood vessels [46]. An in vitro study conducted by Cimmino et al. clearly demonstrated that ROS production in ECs results in the development of a pro-coagulant state through the increase of Factor III which directly binds to the activated cofactor VII, leading to subsequent activation of factors IX and X [47]. Furthermore, is it known that NO plays key roles in vascular homeostasis and in ECs. ROS overproduction provokes the reaction of NO with superoxide anion (O_2_^●−^), leading to the formation of the much more powerful oxidant peroxynitrite (ONOO^−^) which, in turn, leads to serine-protease and fibrinogen nitration [48]. The nitration of fibrinogen promotes subsequent establishment of a pro-coagulant environment, resulting in a fibrin clot [48]. Besides the increase and dysregulation of coagulation cascade factors, thrombosis occurs following a decrease in anticoagulant factors, such as protein S, a cofactor of protein C, whose activation is able to inactivate factors V and VIII of the hemostasis cascade. Antithrombin 3 is another anticoagulant factor that inhibits several proteases, including procoagulant factors II and X [49] (Figure 2).

### 2.3. Fibrinolysis 

Fibrinolysis constitutes the last phase of hemostasis and consists of plasminogen (PA) activation to produce plasmin, which is induced by different serine-proteases, such as the antecedent plasmatic thromboplastin factor (Factor XIa). The latter activates Factor IX as well as the Hageman Factor which, in turn, leads to the activation of Factors XI, VII, and V. The plasmin produced is able to promote the lysis of fibrin, accelerating clotting degradation [50]. However, a relative increase in the plasmin concentration is avoided through the actions of plasminogen activator inhibitors (PAI-1 and PAI-2) and the thrombin activator fibrinolysis inhibitor (TAFI) [51]. The alteration of the fibrinolytic system in ECs could be caused by ROS, which are able to upregulate PAI-1, which is also implicated in the development of the atherothrombotic process. Accordingly, PAI-1 represents a potent inhibitor of fibrinolysis that plays a pivotal role in the pathogeneses of different diseases with greater cardiovascular risks [52]. An interesting study evaluated the effects of ROS in PAI-1 upregulation related to cytokine activation in ECs transfected with plasmid p800LUC, which contains a fragment of the PAI-1 promoter. The authors showed that PAI-1 promoter activity was significantly increased in the presence of a high concentration of ROS and, conversely, was reduced by 75% following treatment with antioxidant agents [53]. Another in vitro study investigated the role of NOXs, specifically NOX-4, in the regulation of PAI-1 expression in human umbilical vein endothelial cells (HUVECs) [53]. Using the specific knockdown of NOX-4, obtained by silencing mRNA (siRNA), a significant reduction in ROS production and, consequently, in PAI-1 release and activity was observed in HUVECs [54]. There is experimental evidence showing that PAI-1 expression is strongly increased during the inflammatory process and during atherothrombogenesis, and previous studies have shown that the oxidation of LDLs, with consequent formation of ox-LDLs, induces the overproduction and release of PAI-1 in ECs. In accordance with these findings, there is evidence that NOX expression induced by ox-LDLs could induce a reaction cascade starting from ROS overproduction and leading to upregulation of the PAI-1 gene [55] (Figure 2).

## 3. ROS and Endothelial Dysfunction 

It is widely accepted that the maintenance of the structure and the functionality of the endothelium are crucial in vascular homeostasis, and there is experimental evidence showing that ROS and RNS overproduction is responsible for endothelial dysregulation. Additionally, disturbance of endothelial function is considered the first major event in atherogenesis.

The ability of ECs to produce O_2_^●−^ through the actions of different isoforms of NOX represents a crucial mechanism that could lead to the overproduction of free radicals [56,57] ECs as well as fibroblasts and vascular SMCs express NOX-1, NOX-2, NOX-4, and NOX-5. In particular, NOX-1 and NOX-2 are involved in the development of hypertension, inflammation, and endothelial dysfunction [58]. The NOX-5 isoform is calcium-dependent and its concentration is generally increased in response to the ROS-induced damage mechanism in atherosclerosis [56,59]. The NOX-4 isoform is able to regulate several pathways including those involved in the signaling of insulin [60] and the differentiation of cardiac cells [61]. Physiological levels of NOX-4 have been implicated in the maintenance of vascular tone, inflammatory stress, and ischemia [62]; however, when NOX-4 levels are increased, high levels of H_2_O_2_ and O_2_^●−^ production have been registered [63]. Consequently, the production of free radicals induced by the different isoforms of NOX influences the activity of other enzymes such as NOS, producing NO [64]. NO is a highly reactive gaseous radical that is able to diffuse across cell membranes [65]. The generation of NO is a product of oxidation of the terminal guanidino nitrogen of l-arginine and is catalyzed by three major isoforms of NOS through a highly regulated process: the calcium/calmodulin dependent NOS isoforms, expressed constitutively (cNOS) in endothelial cells (eNOS) and brain tissue (nNOS), and the cytokine-inducible and calcium/calmodulin-independent form named iNOS [66]. Several studies have reported that, in ECs, the endogenous production of NO at nanomolar concentrations through eNOS activation represents a vasoprotective molecular mechanism of the vascular endothelium, while exaggerated release of NO as a consequence of iNOS activation leads to the rapid reaction of NO with O_2_^●−^, generating ONOO^−^, the main compound responsible for the onset of endothelial dysfunction and, in the late stages, the development of atherothrombosis [67,68,69].

In particular, the uncoupling of eNOS, which represents a crucial mechanism in the development of endothelial dysfunction and increases the risk cardiovascular disease, leads to a reduction in NO production and increased release of RNS, further contributing to the onset of atherothrombosis [70]. In particular, ROS activity leads to significant NO degradation caused by its reaction with O_2_^●−^. In addition, the conversion of eNOS removes its ability to produce NO and, instead, O_2_^●−^ is formed. This event is related to the oxidation of the tetrahydrobiopterin (BH4) cofactor, with subsequent depletion of L-arginine, leading to the accumulation of endogenous methylarginase. Several studies have shown that this mechanism is a crucial mediator of endothelial dysfunction [71,72]. An in vivo study using an hyperphenylalaninemic (hph-1) mouse model, in which there is an important deficiency of BH_4_, demonstrated that animals aortic endothelial cell relaxation due to the uncoupling of eNOS and abnormal downstream regulation due to its dysfunction [73,74]. It is well known that endogenous antioxidant enzymes, such as the different isoforms of SOD, are able to prevent the pathological reduction of NO and ROS overproduction in mitochondria, particularly at the levels of the respiratory chain complexes I and III. Evidence suggests that an imbalance between the activity of antioxidant enzymes, such as glutathione peroxidase (GPx), catalase, and SOD, and the pro-oxidant system produces an uncontrolled increase in oxidative stress, leading to endothelial dysfunction [75].

## 4. Autophagy in Endothelial Cell Biology

Autophagy is a well-studied and critical process for endothelial homeostasis. Indeed, the autophagy process, as a physiological pattern, represents an important tool that provides selective clearance of damaged organelles, protein aggregates, dysfunctional ribosomes, and lipid droplets, which clears the cells of potentially toxic byproducts and allows the recycling of organelle components for bioenergetics [76]. Autophagy represents a protective process associated with cell survival, particularly during when cells are in a low nutritional or starvation state [76]. Furthermore, autophagic flux is involved in the paracrine regulation of vasoactive substances from the endothelium, the induction of vascular SMC switching from a contractile to a proliferative phenotype following injury, and the inhibition of angiogenesis and vascular calcification, thereby promoting endothelial function [23,77]. The induction of autophagosome formation seems to be involved in the mediation of mechanical stimulation and is necessary to allow cells to gradually respond to mechanical stress. These stimuli involve mammalian target of rapamycin (mTOR)-dependent autophagic activation, allowing cells to adapt to the various changes they undergo [78]. In this regard, blood flow through the endothelium generates mechanical force as shear stress, leading to the modulation of gene expression, proliferation, migration, permeability, thrombogenicity, and inflammation [79]. Interestingly, laminar shear stress modulates the endothelial structure through the activation of autophagy. This action is associated both with the phosphorylation of eNOS, in order to produce NO, which subsequently decreases oxidative stress and inhibits inflammatory cytokine production [80], and the inhibition of vasoconstriction mediated by endothelin-1 [81]. On the contrary, the loss of this mechanism in autophagy-deficient cells leads to ROS production and an inflammatory response [80]. The central role of eNOS is also highlighted by evidence that endothelial autophagy modulates eNOS uncoupling through caveolin-1 (Cav1). Indeed, Cav1, the major structural protein of caveolae in endothelial cells, is a modulator of the autophagic process and has a scaffold domain for eNOS binding, inhibiting its activity [82]. Further evidence of the positive role of autophagy in ECs is that the blockade of microtubule-associated protein light chain 3 (LC3)-I (cytosolic form of LC3)/LC3-II (LC3-phosphatidylethanolamine conjugate) protein suppression, induced by ionizing radiation, determines cell survival [83]. These data are supported by the observation that autophagy protects ECs from high-glucose-induced injury [84], as has also been reported in diabetic patients, in whom the restoration of autophagy was found to preserve endothelial function, promoting eNOS activation, thus confirming that intact autophagy is necessary for eNOS signaling [85]. Furthermore, autophagic induction protects human umbilical vein ECs (human umbilical vein endothelial cells) exposed to a high glucose content, promoting tubulogenesis and cell survival activity [86]. Importantly, autophagic flux also preserves the endothelium from angiotensin-II-induced damage [87]. Recent evidence has shown a protective role of antioxidants, such as quercetin or resveratrol, in ECs through the enhancement of the autophagic pathway. In particular, quercetin is able to preserve HUVECs from high glucose damage by reducing the level of ubiquitin-binding protein (p62), an autophagy substrate recognized as a marker of defective autophagy, and by inducing the production of Beclin-1 and subsequent upregulation of the LC3-II to LC3-I ratio [88]. Resveratrol attenuates endothelial oxidative damage by inducing autophagy via activation of the transcription factor EB (a dominant regulator of the autophagy–lysosome pathway), increasing LC3 production and favoring p62 degradation and autophagosome formation [89]. Autophagy also mediates communication between the endothelium and ECM, which is important for the growth, survival, and mitigation of ECs [90]. Indeed, soluble-matrix-derived constituents, such as decorin, collagen VI, kringle 5, and endostatin, act as inducers of the autophagic process, triggering autophagy in a Vps34/beclin 1/LC3-dependent manner and reducing Wnt/β-catenin signaling. Mediation of this pathway is necessary for the protein–protein interactions that form microfilaments that are essential for the support of the surrounding tissue (collagene IV) and is involved in the induction of vascular endothelial growth factor receptor 2 (VEGFR2) (decorin). Moreover, it has been shown that the autophagic pathway can mediate antiproliferative and antimigratory effects (endostatin) through Wnt/β catenin signaling or angiostasis (endorepellin) effects. [91]. The modulation of angiogenesis through autophagic regulation of vascular endothelial growth factor (VEGF) has been demonstrated in cultured bovine aortic endothelial cells (BAECs). Indeed, the stimulation of autophagy with Atg5 resulted in an increase in angiogenesis induced by VEGF. In contrast, the use of 3-methyladenine, an autophagy inhibitor, was found to have an anti-angiogenic effect [92]. Moreover, rapamycin-mediated inhibition of mTOR protects the vascular system from aging by reducing oxidative stress. In addition, mTOR inhibition promotes 5′ adenosine monophosphate-activated protein kinase (AMPK) activation and increases the expression of proteins involved in cell cycle control [93]. There is evidence of the involvement of autophagy in the secretory role of endothelium. Indeed, ECs release the so-called Weibel–Palade bodies (WPBs), which are necessary for the adhesion of platelets to the injured vessel walls [94]. WPBs have been implicated in the autophagic process, as they have been recorded near or within autophagosomes, and the von Willebrand factor (vWF) is secreted within WPBs. Interestingly, it was shown that genetic or pharmacological inhibition of autophagic mediators, such as Atg5 or Atg7, is associated with an increase in the p62 level and a decrease in the LC3II level, resulting in reduced vWF secretion in HUVECs. Furthermore, in a murine model characterized by an endothelial-specific deletion of Atg7, despite a normal vessel architecture and capillary density, impaired epinephrine-stimulated vWF release was registered, resulting in a prolonged bleeding period. Finally, the authors demonstrated that the endothelial knockdown of Atg5 or the pharmacological inhibition of autophagy resulted in a similar in vivo alteration of hemostasis [95] Accordingly, Atg7 deletion is associated with a reduction in arterial thrombosis in a murine model of atherothrombosis [96]. Moreover, Atg5 interaction is necessary to promote n-3 polyunsaturated fatty acid (n3 PUFA)-induced atheroprotection [97]. Recently, autophagy has been proven to be an important tool in the differentiation of stem cells into endothelial lineage by promoting angiogenesis through the release of proangiogenic factors in a paracrine manner [98]. Indeed, it was shown that autophagy in human bone marrow monocytic cells is accompanied by the expression of specific markers, such as FLK-1, Tie-2, Cadherin-12, and Cadherin-19, after the induction of monocyte chemotactic protein-1 [99]. Moreover, in ECs, the autophagic process significantly supports haematopoietic stem cell homeostasis. Indeed, the impairment of bone marrow ECs is prevented by the modulation of Beclin-1 signaling, which activates autophagy in patients with poor graft function [100].

Overall, the activation of autophagy mediators in vascular biology is necessary for the physiological changes that occur after blood flow during the adaptation of the vascular endothelium and for the growth and survival of endothelial cells, promoting angiogenesis and differentiation. Moreover, on this basis, an in-depth analysis of the relationship between autophagy and agents involved in the modulation of this important process is necessary to better understand when autophagy is promoted or inhibited, allowing these notions to be translated into the clinical field.

## 5. Crosstalk between Oxidative Stress and Autophagy 

In recent years, several findings have supported the roles of ROS and NOS in intracellular signaling, which mediates autophagy upon nutrient deprivation [101,102]. Indeed, endogenous overproduction of ROS/RNS represents the main biological source of highly reactive oxidant molecules that irreversibly damage cells, whereas basal protective autophagy represents an efficient repair system that contributes to the removal of damaged intracellular organelles and macromolecular aggregates and simultaneously generates an endogenous nutrient pool of macromolecules to sustain metabolic needs [16]. 

One main question is how ROS/RNS drive the autophagic process and which are the main free radical species involved. 

Some studies have identified O_2_^●−^ as the primary source of ROS involved in autophagy induced by nutrient starvation [103], while others have recognized H_2_O_2_ as the first molecule produced immediately after starvation [104]. However, this still remains to be clarified and most of the scientific research refers to ROS as a whole as inducers of autophagy [105]. ROS can induce autophagy by mediating various signaling pathways during the process of autophagosome formation. In the induction stage, ROS can induce autophagy by inhibiting phosphatidylinositol 3-kinases (PI3K)-protein kinase B (PKB/Akt)-mTOR [106]. In particular, it was reported that ROS specifically induce AMPK activation, which was able to inhibit the activity of the mTOR signaling pathway in an experimental animal model of cardiovascular damage [107]. Moreover, under starvation conditions, cells produce large amounts of ROS, particularly H_2_O_2_, which was found to mainly oxidize and inhibit Atg4 activity, causing LC3-II accumulation and thus promoting autophagosome formation. In addition, ubiquitinated materials combine with p62 and LC3-II to form targets of autophagic bodies and are degraded [108,109]. Other studies have found that ROS can contribute to activation of the mitogen-activated protein kinase (MAPK) pathway, which leads to modulation of the activity of the transcription factors activator protein 1 (AP-1), forkhead box transcription factor O (FoxO), and nuclear factor kappa B (NFkB), which, in turn, regulates autophagy-related gene expression, affecting autophagy [110]. There is evidence of the ability of ROS to induce autophagy through the c-Jun N-terminal kinase (JNK) signaling pathway in an in vitro model of mouse mesenchymal stem cells [111]. Several studies have shown that JNK mediates the occurrence of autophagy by activating Atg7 in several in vitro models. Conversely, knockout of the autophagy-related gene Atg7 significantly reduces the H_2_O_2_-mediated autophagy response under oxidative stress conditions. Further studies have suggested that ROS-dependent JNK and extracellular signal-regulated kinase (ERK) activation represent important upstream regulatory mechanisms that are capable of increasing H_2_O_2_ production, thus improving autophagic flux [112]. Notably, autophagosome/lysosome fusion is modulated by ROS production through the involvement of the p38 MAPK signaling pathway and is associated with the gene expression of Atg7 and E3 ubiquitin ligase during protein ubiquitination. In turn, this is associated with FoxO transcription activation [113]. Interestingly, autophagosome formation can be affected by the modulation of Atg4 activity exerted by intracellular redox processes. A study demonstrated that under starvation conditions, several signaling pathway molecules, including PI3K III and Beclin 1, trigger the production of ROS, especially H_2_O_2_. The latter can oxidize Atg4, inhibiting LC3-II delipidation. However, Atg4 can further process the C-terminal residue of LC3, ensuring the formation of autophagosomes [108]. Ruth Scherz-Shouval et al. found that Atg4 contains two major catalytic sites that are critical for its redox regulation. In an experiment conducted in vitro, mutations at these sites significantly affected the sensitivity of the protein to H_2_O_2_, leading to the inhibition of autophagosome formation [109].

Several studies have reported that mitochondria, as the main site of ROS production, are primarily involved in autophagy induction. Under conditions of nutrient deprivation, an increasing energetic deficit occurs, in turn increasing ATP demand. This unbalanced energetic condition leads to mitochondrial overload, which triggers electron leakage and an increase in ROS production [114,115]. Another hypothesis identifies Hexokinase-II (HKII) as a positive regulator of the upstream autophagic signal through inhibition of the activity of target of rapamycin kinase complex 1 (TORC1) on the permeability transition pore, leading to mitochondrial ROS overproduction [116]. In accordance with this evidence, it was reported that Akt and myotonic dystrophy protein kinase (DMPK) upregulate HKII action and negatively modulate autophagy [117,118]. When cells undergo prolonged starvation, chronic impairment of mitochondrial function occurs. Subsequent highly extended production of ROS leads to a shift from bulk autophagy into mitophagy, a more specific self-removal mechanism in which autophagy eliminates the source of oxidative stress, protecting cells from damage. It was recently reported that the main molecular mechanism underlying mitochondrial degradation through mitophagy is regulated by PTEN-induced putative kinase 1 (PINK1) and the ubiquitin E3 ligase Parkin [119,120]. Mitochondrial depolarization induces the translocation of PINK1 to the outer mitochondrial membrane to recruit Parkin which, in turn, ubiquitylates voltage-dependent anion channel 1 (VDAC1) [121]. The ubiquitin-binding protein p62 recognizes the ubiquitylated proteins and triggers their degradation process through the proteasome or the lysosome pathway via autophagy [122]. 

Interestingly, it was found that p62 mediates mitophagy thanks to its LC3-interacting region (LIR) through which the autophagy-targeted mitochondria are connected with LC3 located on the autophagosome surface, allowing their degradation [123]. A recent study identified Ambra1 as another major inducer of mitophagy, regardless of the actions of Parkin and p62 described above [124]. The induction of autophagy upon ROS production from the mitochondria is mediated by the redox-sensitive protein AMPK, which is activated by the s-glutathionylation of reactive cysteine following H_2_O_2_ exposure [125].

The redox-based signals generated by ROS/RNS via the reversible modification of proteins at the level of the sulfur-containing residues cysteine and methionine are known to affect protein structure and function [126]. 

Although it is not considered the main mechanism linking ROS and autophagy, the importance of post-translational oxidative modification in autophagy induction is supported by experimental evidence. This involves the homeostasis and modification of thiol groups. Indeed, under conditions of starvation, cells actively extrude glutathione (GSH) through multidrug resistance protein 1 (MRP1) in order to shift the intracellular redox environment towards more oxidizing conditions and trigger the oxidative modification of redox-sensitive proteins [127]. These notions are supported by evidence that some proteins involved in the autophagic flux, such as the Atg7-Atg3 and Atg7-Atg10 complexes, act through Cys residues. Additionally, p62 has a zinc-finger motif (ZZ) that is rich in cysteine residues which can undergo redox regulation, possibly leading to detrimental oxidation and structural alterations that modulate its role in autophagy [128]. In this regard, an interesting study clearly demonstrated that the oxidation of Atg4 at Cys, mediated by H_2_O_2_, is able to inhibit hydrolyzing activity on LC3, allowing elongation of the autophagosome [109].

On the other hand, the conflicting role of nitrosative stress in relation to autophagy modulation is a complex field of study. Recent studies have suggested that NO, through the S-nitrosylation reaction, has an inhibitory role in autophagosome synthesis via different mechanisms. Indeed, it was clearly demonstrated that treatment with NO donors or the upregulation of NOS activity inhibits autophagy in vitro. In particular, NO impairs autophagy activity by inhibiting the substrates of the S-nitrosylation reaction: JNK1 and inhibitor of nuclear factor kB kinase β (IKKβ). JNK inhibition results in the reduction of Bcl-2 phosphorylation which, in turn, increases Bcl-2–Beclin 1 interactions, disrupting the formation of the hVps34/Beclin 1 complex. In addition, IKKβ inhibition reduces AMPK phosphorylation, leading to mTORC1 activation via tuberous sclerosis complex 2 (TSC2) [129]. In accordance with these results, some other experiments have shown that *S*-nitrosyation reactions downregulate TSC2 inhibitory activity on mTOR, avoiding autophagy activation [130].

## 6. The Critical Role of Autophagy in the Development of Atherosclerosis: Focus on Endothelial Cells

**Figure 3:**
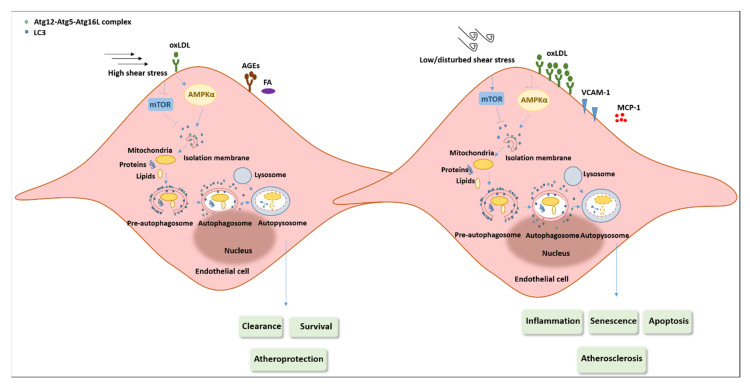
Role of autophagy in endothelial cells during atherosclerosis. Different atherogenic stimuli, such as oxLDLs, AGEs, and saturated FA, can stimulate autophagy/mitophagy in ECs. Additionally, exposure to high shear stress can promote protective autophagy in ECs, playing antiapoptotic, antisenescent, anti-inflammatory, and antiatherogenic roles. Conversely, the exposure of ECs to low and disturbed levels of shear stress as well as oxLDL accumulation impairs autophagic flux as a result of inhibition of the AMPKα pathway and activation of the mTOR pathway as well as the blockade of fusion between autophagosomes and lysosomes. Defective autophagy leads to hyperactivation of inflammasomes, defective cholesterol efflux, and senescence and apoptotic cell death, thus exacerbating the atherosclerotic process. Solid lines indicate upregulated pathways. Dashed lines indicate downregulated pathways. AGEs: advanced glycation and products; ECs: endothelial cells; FA: fatty acids; MCP-1: monocyte chemoattractant protein-1; oxLDL: oxidized low-density lipoproteins; VCAM-1: vascular cell adhesion protein-1.

The contribution of oxidative stress to atherosclerosis development is well-known, and a large body of evidence indicates that ROS production represents the main upstream intracellular signal transducer that sustains the autophagic flux. It is most likely that autophagy, when stimulated by stress-related stimuli, protects ECs and also SMCs against cell death in the arterial wall. Moreover, it an association between excessive oxygen radicals and defective autophagy has clearly been shown, and this plays a key role in the onset and development of the atherothrombotic process. Thus, the role of autophagy in atherosclerosis seems to be complex, with both protective and detrimental effects at the site of injury, mainly depending on the oxidant concentration and time of exposure as well as on the types of cells involved. There is a growing body of evidence showing that basal levels of autophagy are able to protect vascular cells against oxidative stress and properly mediate vascular function; however, major ROS overproduction can lead to defective autophagy, resulting in the inhibition or hyperactivation of autophagic flux in all major cell types (macrophages, SMCs, and, in particular, ECs) involved in atherosclerotic plaque formation [131]. Thus, excessive ROS production, which leads to pro-oxidative/antioxidative cellular imbalance, and atherosclerotic disease progress in parallel. Accordingly, experimental evidence shows the atheroprotective activity of basal autophagy during the early stages of atherosclerosis development, which becomes dysfunctional in the advanced stages of the disease, triggering cell death, reduced synthesis of collagen, thinning of the fibrous cap, plaque destabilization, and thrombosis [132] (Figure 3). 

Thus, cells react to ROS injury by orchestrating a stress response to prevent further damage. This happens via activation of the autophagic process as well as through the activation of other intracellular proteolytic systems and through increased levels of antioxidant agents. In the first stages of oxidative injury at least, autophagy can remove damaged intracellular components through its fusion with lysosomes, which represent one of the main targets of ROS. Indeed, some in vivo and in vitro studies have demonstrated that high levels of and/or chronic exposure to ROS alter the autophagic process and trigger apoptotic cell death by directly disrupting the structure of lysosomal membranes, rendering them unable to fuse with autophagosomes. This phenomenon induces the release of lysosomal hydrolases into the cytosol, which damage proteins and organelles and, in turn, leads to the activation of the caspase pathway [17]. Moreover, the autophagic process, which is linked with severe oxidative stress, contributes to ceroid formation. This insoluble complex of proteins, associated with oxidized lipids and present in advanced atherosclerotic lesions, accumulates in lysosomes. This condition induces the production of lysosomal enzymes in an unsuccessful attempt to facilitate ceroid degradation, resulting in them losing their active role in autolysosomes, promoting autophagy impairment and apoptosis induction [133]. Failure of the autophagic process, in turn, stimulates further accumulation of damaged mitochondria, increases ROS generation, and enhances the formation of ceroid-containing lysosomes, exacerbating the damage [133].

### 6.1. The Modulation of EC Autophagy in the Onset and Development of Atherothrombosis

During the past few years, the role of autophagy in the pathogenesis of atherosclerosis has been investigated by several research groups. In particular, the EC autophagic process is complex and affects both normal vascular homeostasis and disease. Therefore, studies related to the evaluation of the potential role of autophagy during the modulation of endothelial function and the explanation of how specific deletions of autophagic machinery affect EC function during the development of atherosclerosis are of great importance. 

Evidence suggests that the occurrence of high concentrations of circulating oxy-LDLs is associated with altered reactive vasodilatation, which represents the early stage of endothelial dysfunction [68,134]. It has been hypothesized that oxy-LDLs suppress the release of the constitutive mediator of ECs, NO, through direct or indirect inhibition of eNOS. This, in turn, triggers the activation of iNOS and subsequent toxic effects due to the generation of ONOO^−^, which is responsible for EC damage [69]. 

In this condition, among the mechanisms involved in oxLDL-related endothelial dysfunction and inflammation of vascular tissues, the autophagic pathway plays a pivotal role.

EC autophagy is primarily involved in atherosclerotic plaque formation, and this occurs to protect cells from oxidative stress by degrading multifunctional materials, especially polarized mitochondria. Autophagic engulfment of damaged mitochondria has been shown to limit cytochrome C release, preventing apoptosis and contributing to cell recovery [26]. It was observed that oxidative stress, and in particular mitochondrial ROS production, which occurs in the atherosclerotic environment, leads to autophagy in human ECs. This was shown by the upregulation of Beclin-1 and the lipidated form of LC3, both recognized as markers of autophagosome formation, through AMPK and mTOR signaling activation [135]. A number of studies have demonstrated the close association between increased activity of the autophagic process and lipid degradation in ECs exposed to Ox-LDLs. A study conducted on primary ECs showed a significant accumulation of both native or Ox-LDLs that are engulfed within autophagic structures. To confirm that autophagic activity represents a major mechanism that regulates excess and exogenous lipid levels, transient knockdown of the essential autophagy gene Atg7 was performed. The result, indeed, showed higher levels of intracellular native LDLs and Ox-LDL accumulation in ECs [136]. To support these results and to confirm the critical role of autophagy in limiting lipid accumulation within the endothelium, an in vivo experiment using mice containing a conditional deletion of Atg7 was conducted. The acute intravenous infusion of fluorescently labelled oxLDLs identified a sustained retention of ox-LDLs within the endothelium. Moreover, in apolipoprotein E-deficient (apoE^−/−^) mice, a chronic model of excess lipids, inhibition of endothelial autophagy markedly increased the atherosclerotic burden [136]. Moreover, in EA.hy926 ECs, oxLDL exposure resulted in stronger autophagy features including the remodeling of actin filaments, the rupture of adherent junctions, and autophagosome formation with the characteristic double membrane at the ultrastructural level. In addition, advanced oxLDL exposure times led to the identification of autophagic vacuoles and autophagolysosomes associated with a decrease in the lysosome concentration. These results were accompanied by a significantly increased expression level of LC3-II, reduced signs of apoptosis, and modulation of lectin-like oxLDL receptor-1 (LOX-1) expression [137].

Different genetically engineered mouse models of atherosclerosis have been used to clearly confirm the cytoprotective role of autophagy. In a series of studies, Atg5 knockdown was shown to trigger detrimental proatherosclerotic activity characterized by the induction of inflammasomes and apoptosis, subendothelial formation of cholesterol crystals, defective or inefficient efferocytosis, and impaired cholesterol efflux from macrophages [138,139]. Accordingly, the deletion of Wip1 phosphatase, an mTOR-dependent inhibitor of autophagy, is able to reduce the development of atherosclerotic plaques and positively modulate lipid metabolism [140]. An interesting study published in Scientific Reports showed a differential role of autophagy depending on the cell type in atherosclerotic development [141]. The data showed that oxLDLs inhibit the activity of mTOR and thus upregulate the protein level of Atg13 and its dephosphorylation, activating the autophagic pathway as a whole in HUVECs. In vivo experiments have reported that mTOR activation decreases the protein level of Atg13 in the plaque endothelium of apoE^−/−^ mice, whereas the activities of mTOR and autophagy in the macrophage cell line RAW246.7 and the vascular SMCs of apoE^−/−^ mice are not affected. These results suggest that the inhibition of autophagy through mTOR activation protects the endothelium and stabilizes atherosclerotic lesions [141]. Other studies have reported that, similarly to macrophages and SMCs, excessive autophagy activation mediates autophagic-induced cell death in ECs, provoking plaque instability [142]. Vion and colleagues recently showed that defective autophagy contributes to the development of atherosclerotic plaques, promoting inflammation, apoptosis, and a senescent phenotype in ECs [143]. Several experiments have revealed that the apoptotic death of ECs occurs when the autophagic pathway is over-activated, leading to plaque structure destabilization, increased inflammation, and thrombosis formation [144]. In this regard, autosis has recently been defined as a new form of cell death triggered by autophagy, which is characterized by increased cell–substrate adhesion, focal swelling of the perinuclear region, and disassociation of the endoplasmic reticulum with impaired membrane Na^+^/K^+^-ATPase activity [145]. The critical role played by the autophagic process, which is dependent on the oxidative state and on the severity of the atherosclerotic process, has been further evaluated [146]. An in-depth study demonstrated that in HUVECs exposed to 20 to 40 μg /mL of ox-LDL, the autophagic process was activated, as shown by significant increases in the Beclin-1 expression level and the lipidated form of LC3 [147]. However, a higher concentration of ox-LDL (60 μg/mL) decreased the levels of both autophagy markers, suggesting that autophagy reflects a stress response that allows cells to survive under pathological conditions. In addition, it was shown that the autophagic response is mediated by the ROS–LOX-1 pathway in HUVECs [147]. The authors also identified strong autophagic activation, toll-like receptor 9 (TLR9) expression, and inflammatory signals in the aortas of LDLR knockout mice fed a high-cholesterol diet. In these animals, the deletion of LOX-1 (LDLR/LOX-1 double knockout mice) attenuated autophagy, TLR9 expression, as well as the inflammatory process [147]. Mollace and colleagues also hypothesized that a crucial role is played by the scavenger receptor LOX-1 in the modulation of autophagic flux in BAECs exposed to oxLDL (1–100 μM) [148]. The authors showed that after 48 h of incubation, increased concentrations of oxLDL induced proportional increases in malondialdehyde (MDA) production and apoptotic cell death in treated BAECs. The detrimental effect of oxLDLs, mediated by LOX-1 overexpression, was found to attenuate the protective autophagic response, which was detected by the expression levels of Beclin-1 and LC3II and became consistent at the highest concentration of oxLDL used. Moreover, in vitro silencing of LOX-1 receptor via shRNA was shown to significantly restore LC3 expression in 100 μM of oxLDL-treated BAECs, thus suggesting that LOX-1 overproduction plays a pivotal role in oxyLDL-induced endothelial dysfunction, which characterizes the early stages of the atherosclerotic process [148]. An animal model studied by Razani and colleagues confirmed that autophagy becomes dysfunctional during atherosclerotic plaque progression in apoE^−/−^ mice fed a Western-type diet. In this study, the p62 level was dramatically increased in atherosclerotic aortas and this further increased with age and plaque burden [138].

Using young and replicative human senescent ECs as well as different animal models of atherosclerosis (apoE^−/−^, Arg2^+/+^ and Arg2-deficient apoE^−/−^), an in-depth report published on autophagy demonstrated the role of Arginase-2 (ARG2) and the mechanisms potentially underlying the modulation of endothelial autophagy [149]. The authors showed that the overexpression of ARG2 impairs endothelial autophagy through the activation of Ribosomal Protein S6 Kinase B1 (RPS6KB1) and inhibition of the protein kinase, AMP-activated, α catalytic subunit (PRKAA), which is implicated in atherogenesis, whereas the silencing of RPS6KB1 or expression of constitutively active PRKAA prevented autophagy suppression in young cells. Conversely, enhanced ARG2-RPS6KB1 and decreased PRKAA signaling and autophagy were observed in senescent cells [149]. Another interesting report published on Autophagy in 2018 highlights the close relationship between autophagic flux and shear stress within the endothelium and its role in atherosclerosis development [150]. In brief, the presence of defective autophagy was observed in low shear stress areas, which is responsible for the preferential plaque formation in these areas. In contrast, areas of high shear stress induce autophagy activation, protecting ECs from inflammation, senescence, apoptosis, and atherosclerotic development. In high shear stress areas of murine and human arteries, increased numbers of endothelial autophagosomes and LC3 puncta were identified. This result was confirmed in cultured ECs exposed to high shear stress. Moreover, it was demonstrated that a low level of shear stress is associated with increased mTOR activity and decreased AMPK activity, which are both inversely modulated under high shear stress conditions. Using hypercholesterolemic mice bearing an endothelial specific deletion of Atg5 (apoE^−/−^; Atg5flox/flox; Cdh5/VE-cadherin-Cre), the authors reported that even in normally atheroprotected areas exposed to high levels of shear stress, defective autophagy leads to larger plaque sizes. Finally, defective autophagy was associated with an increase in the inflammatory process in cultured ECs stimulated with TNFα in the presence of a high level of shear stress. In mice deficient in endothelial Atg5, the atheroprone areas are characterized by an increased number of TUNEL- and transformation-related protein 53 (TRP53)/p53-positive nuclei, suggesting a pro-senescent and pro-apoptotic endothelial phenotype [150] (Table 1). 

### 6.2. Crosstalk between Non-Coding RNAs and Autophagy in the Atherosclerotic Process

MicroRNAs (miRNAs) have emerged as critical modulators of EC function and play vital roles in several biological processes [151]. In particular, miRNA-126 is an EC-specific miRNA and a critical regulator of the atherosclerotic process. It has been shown that the overproduction of oxLDLs significantly inhibits miRNA-126 levels and triggers impaired autophagic flux in HUVECs [152].

In particular, the authors showed significantly increased levels of beclin-1, LC3II/LC3I, and also p62, which is usually degraded by normal autophagic flux, after ox-LDL treatment. Furthermore, to clarify whether the increases in beclin-1 and LC3II/LC3I are related to an increase in autophagic initiation or a decrease in autophagic degradation, the HUVECs were pre-treated with bafilomycin A1, which inhibits the merging of autophagosomes and lysosomes. The data showed that the increased protein levels of LC3-II/LC3I, Beclin 1, and p62 were not further significantly increased in response to bafilomycin A1 pretreatment and oxLDL treatment, suggesting a defect in autophagy flux due to inhibition of the autophagic degradation process. Interestingly miRNA-126 overexpression induced by miRNA-126 mimics remarkably inhibits oxLDL-induced HUVEC injury and restores impaired autophagy flux, characterized by increases in beclin-1 and LC3II/LC3I and a significant reduction in p62 expression. Furthermore, the restoration of autophagy flux, promoted by miR-126 overexpression, was correlated with suppression of the PI3K/Akt/mTOR pathway [152]. Moreover, an interesting study identified miRNA-216a as a molecular link between autophagy and endothelial dysfunction. The authors demonstrated an inverse correlation between miRNA-216a and autophagic genes in HUVECs subjected to aging and in vivo models of human atherosclerosis plaques obtained from patients who underwent carotid endarterectomy, identifying Beclin-1 as a direct target of miR216a. They showed that the overexpression of miRNA-216a in young HUVECs inhibits the expression levels of beclin-1 and Atg5 and consequent oxLDL-induced autophagy, leading to ox-LDL accumulation and monocyte adhesion, as evaluated by an analysis of LC3. Conversely, miRNA-216a inhibition allows the induction of protective autophagy in response to an oxLDL stimulus in old HUVECs [153]. Accordingly, recent findings showed the involvement of miRNA-214-3p in EC autophagy regulation of atherosclerosis [154]. The authors reported that miRNA-214-3p is inversely correlated with Atg5 and Atg12 in CD31+ endothelial cells from the aorta of apoE^−/−^ mice and in young HUVECs transfected with miRNA-214-3p mimics/inhibitors and stimulated with 100 µg/mL of ox-LDL. In brief, the overexpression of miRNA-214-3p inhibits ox-LDL-induced autophagy, as assessed by marked reductions in Atg5, Atg12, and LC3B-II protein expression levels and a decrease in the p62 expression level. These results are associated with the reduced formation of endogenous LC3B-II punctiform, oxLDL accumulation, and monocyte adhesion, promoting a significant decrease in cell viability. On the contrary, the suppression of miRNA-214-3p preserved the ability to initiate a protective autophagy reaction under conditions of oxLDL stimulation in old HUVECs [154]. Recently, lncRNAs have gradually attracted the interest of researchers, although their participation in the atherosclerotic process is still unclear. One recent published work revealed that oxLDLs specifically decrease the level of lncRNA-FA2H-2, which probably regulates the concentration of the mixed lineage kinase domain-like protein (MLKL), which plays a crucial role in ox-LDL-regulated autophagy and inflammation [155]. Some in vitro experiments have reported that treatment with oxLDLs leads to the increased expression of VCAM-1, MCP-1, interleukin (IL)-6, IL-8, IL-18, IL-1β, and tumor necrosis factor- α (TNF-α) in a dose-dependent manner. Additionally, the monitoring of autophagic markers revealed high levels of LC3II/LC3I, p62, and lysosomal-associated membrane protein 1 (LAMP1), as well as a reduced level of cathepsin D, a lysosomal protease involved in autophagic degradation. These findings were associated with further accumulation of autophagosomes, mainly due to impaired autophagic degradation in ECs. Interestingly, rapamycin pretreatment leads to LC3II and LAMP1 accumulation but reduces the p62 level.

The silencing of lncRNA-FA2H-2 has been shown to upregulate MLKL by worsening inflammation and inhibiting autophagic flux in ox-LDL-stimulated ECs and SMCs. Such harmful processes have mainly been associated with activation of the PI3K/Akt/mTOR signaling pathway.

Indeed, the protein levels of VCAM-1, MCP-1, and IL-6 induced by ox-LDLs were shown to increase while the levels of LC3II/LC3I and LAMP significantly decreased, as did the number of autophagosomes. Under these conditions, the expression level of p62 was markedly increased and, interestingly, pre-treatment with bafilomycin A1 had no obvious effect on the protein levels of LC3II, p62, and LAMP1. 

Accordingly, in apoE^−/−^ mice fed a Western diet, it was demonstrated that the further knockdown of LncRNA-FA2H-2 decreases LC3II expression and LAMP-1 while enhancing the expression of p62, MLKL, VCAM-1, MCP-1, and IL-6 in atherosclerotic lesions [155] (Table 1). 

**Table 1:** Studies on defective autophagy in endothelial cells: role in atherothrombosis.

In Vitro/In Vivo Model	Results	References
- shAtg7 HUVECs treated with 50 μg mL^−1^ oxLDLs - Atg7^endo^ mice (fl/fl;VE-Cadherin Cre) mice - HFD-fed apoE^−/−^Atg7^endo^ mice	↓ LC3-II/ LC3I, ↑ oxLDLs ↑ intracellular ^125^I-LDL chol ↑ transcytosis intracellular ^125^I-LDL chol ↑ dil-oxLDLs in RPE and choroidal endothelium ↑ atherosclerotic lesion size ↑ necrotic area	[136]
- HUVECs treated with 50 mg/mL oxLDLs - ApoE^−/−^ mice	↓ mTOR, ↑ Atg13, ↓p-Atg13 ↑ apoptosis of endothelial cells ↑ atherosclerotic lesion areas ↑ instability of plague, ↑ lipid deposition, ↑SMCs ↑ macrophage cell area ↑ MMP-2 MMP-9 activity ↑ ICAM-1, VCAM-1; IL-6, IL-8	[141]
- HFD-fed apoE^−/−^Atg5^flox/flox^ mice - HFD-fed apoE^−/−^Atg5^flox/flox^; VE-cadherin-cre mice - Atg5^flox/flox^;VE-cadherin-cre - Atg7^flox/flox^;VE-cadherin-cre mice - wortmannin-inhibited autophagy in HUVECs exposed to high SS - sh-Atg5 lentivirus in HUVECs exposed to high SS - HFD-fed Atg5 or Atg7-deficient mice	↑ plague formation, ↑ plague size in atheroresistant regions ↑ serum glucose levels - disturbed endothelial alignment in response to flow ↓ KLF2, ↑ ICAM-1, and MCP-1 *↑*; *p53*, ↑ p16, ↑ SA-β-gal	[143,150]
- HUVECs treated with 20–40 μg/mL oxLDLs - LDLR knockout mice	↑ Beclin-1, ↑LC3-II/ LC3I, ↑ LOX-1 ↑ mtDNA damage, ↑ TLR9, ↑ CD45 and CD68	[147]
- BAECs treated with 100 μM oxLDLs	↓ Beclin-1, LC3-II/LC3I, ↑ LOX-1 ↑ MDA, ↑apoptosis	[148]
- young HUVECs or HAECs transfected with adenovirus-mediated overexpression of ARG2 or inactive ARG2 (H160F) - HFD-fed apoE^−/−^Arg2^+/+^ mice - HFD-fed apoE^−/−^Arg2^−/−^ mice	↓ LC3II/LC3I, Atg12–Atg5 conjugate, ↑ p62 ↑ SA-β-gal ↓ PRKAA-T172/PRKAA, ↑ RICTOR ↑ AKT-S473/AKT, RPS6-S235/236:RPS6 ↑ TP53-S15/TP53	[149]
- HUVECs treated with 100 μg/mL oxLDLs	↓ miRNA-126, ↓ cell viability ↑ caspase-3 activity, ↑ apoptosis rate, ↑ LDH release - defective autophagic flux and degradation ↑ Beclin-1, ↑ LC3-II/LC3I, ↑ p62, ↑ p-mTOR/mTOR ↑ p-PI3K p85/ PI3K p85, ↑ p-Akt/Akt	[152]
- young HUVECs transfected with miRNA216a - HAECs transfected with miRNA216a - HCAECs transfected with miRNA216a - young HUVECs transfected with iRNA216a and treated with 100 μg/mL oxLDLs	↓ Beclin-1 and Atg5 mRNA and protein levels ↓ LC3II/LC3I, ↑ oxLDL accumulation ↑ monocyte adhesion	[153]
- CD31^+^ aortic endothelial cells derived from HFD-fed apoE^−/−^ mice - young HUVECs transfected with miRNA214-3p and treated with 100 μg/mL oxLDLs	↓ Arg5 and Atg12, ↑ p62, ↓ LC3II ↑ oxLDL accumulation, ↑ monocyte adhesion ↓ cell viability	[154]
- HUVECs treated with 25, 50, or 100 μg/L oxLDLs - HUVECs transfected with si-LncRNAFA2H-2 and treated with 25, 50, or 100 μg/L oxLDLs - HFD-fed apoE^−/−^ mice infected with si-LncRNAFA2H-2	↑ VCAM-1, MCP-1, TNF-α ↑ IL-6, IL-1 β, IL-18, IL-8 ↓ IL-10 - impaired autophagic degradation ↑ LC3-II/LC3I, p62, LAMP1 ↓ cathepsin D ↓ LncRNAFA2H-2, ↑ MLKL ↓ p-PI3K, p-Akt, p-mTOR, ↑ AMPK ↑ lesion areas, proliferative and disarranged intima ↑ lipid-laden foam cells ↑ cholesterol crystals	[155]

↑ increase, ↓ decrease. Atg: autophagy-related protein; HUVECs: human umbilical vein endothelial cells; HFD: high fat diet; apoE^−/−^: apolipoprotein E-deficient; LC3-II: microtubule-associated protein light chain 3-II; ^125^I-LDL: intracellular low density lipoprotein; Chol: cholesterol; dil-oxLDL: fluorescently labelled oxLDLs; RPE: retinal pigment epithelium; mTOR: mammalian target of rapamycin SMCs: smooth muscle cells; MMP: metalloproteinase; ICAM-1: intracellular adhesion molecule 1; VCAM-1: vascular cell adhesion molecule 1; IL: interleukin; KLF-2: Krüppel-like Factor 2; MCP-1: *monocyte chemoattractant protein-1*; SA-β-gal: senescence associated β-galactosidase; SS: shear stress; Akt: protein kinase B; TLR9: toll like receptor-9; LDLR: low density lipoprotein receptor; LOX-1: Lectin-like oxidized low-density lipoprotein receptor-1 BAECs: Bovine aortic endothelial cells; HAECs: human aorta endothelial cells; ARG: arginase; PI3K: *phosphatidylinositol 3-kinases*; LDH: lactate dehydrogenase; HCAECs: human coronary artery endothelial cells; TNF-α: tumor necrosis factor- α; MLKL: mixed lineage kinase domain-like protein; AMPK: AMP-activated protein kinase.

### 6.3. Macrophage Autophagy in Atherosclerosis

Leukocytes are involved in several mechanisms that lead to the development of cardiovascular complications. In the leukocyte population, macrophages are the main type of inflammatory cell that play a key role in the atherosclerotic process [156].

There is evidence to suggest that macrophage autophagy play a protective role in atherosclerosis and in the consequences of impaired macrophage autophagy during the development and progression of atherosclerosis [132]. 

In the early stages of atherosclerosis, the activation of autophagy is able to protect plaque cells from oxidative damage induced by free radicals, oxLDLs, and high cytokine production due to the inflammatory state. Hence, the autophagic process leads to cytoprotection and inhibits the induction of apoptotic cell death. Furthermore, since the generation of foam cells is due to the excessive influx and accumulation of LDL and cholesterol esters in the intimal macrophages, the early autophagic process promotes the clearance of dead cells (efferocytosis) through macrophages and cholesterol efflux from the foam cells [157]. 

However, during conditions of severe oxidative stress, the autophagic process is unable to remove the damaged organelles and, in particular, the insufficient elimination of mitochondria leads to consequent activation of the intrinsic apoptotic pathway. Autophagy failure also leads to the formation of ceroid-containing lysosomes. The interconnection between autophagy and apoptosis modulates the progression and stability of atherosclerotic plaques. 

Another crucial mechanism involved in the impairment of intimal autophagy is the increased production of NO; this is due to the high level of iNOS expressed by macrophages in human atherosclerotic plaques. Indeed, higher NO levels can lead to the reaction of NO with superoxide to produce peroxynitrite which, in turn, leads to tissue impairment due to protein nitrosylation and lipid oxidation [158].

In advanced atherosclerotic plaques, impaired macrophage autophagy is caused by increased levels of iNOS, the aging process, and ceroid deposition. Impaired autophagy results in increased sensitivity to apoptotic stimuli with subsequent production of plaque instability, necrosis, and formation of atherothrombotic lesions [132,133,134,135,136,137,138,139,140,141,142,143,144,145,146,147,148,149,150,151,152,153,154,155,156,157,158]. 

Indeed, an in vivo study conducted by Liao and colleagues investigated the consequences of defective efferocytosis in advanced atherosclerosis in a mouse model of Atg5 depletion. The results demonstrated that the inhibition of autophagy exacerbates atherosclerotic plaque development due to increased apoptosis and ROS-production-related NOX hyperactivity as well as the difficult recognition of apoptotic cells by efferocytes [159]. Another interesting in vivo study using a mouse model of Atg5 depletion suggested that impaired autophagy leads to crystal formation and inflammasome hyperactivation in autophagy-deficient plaques [138]. 

### 6.4. The Link between Autophagy and Ferroptosis in Atherothrombosis

In recent years, in addition to the well-known canonical apoptosis, necrosis, and autophagy-dependent cell death, many other types of cell death have been discovered, including ferroptosis [160]. Ferroptosis is an iron-dependent form of non-apoptotic cell death, marked by a requirement for the accumulation of lipid ROS and characterized by typical morphological, biochemical, and genetic features [161]. Several studies have enriched the knowledge on how ferroptosis occurs, outlining both the generative mechanism involving iron overload and lipid peroxidation, considered to be the two central biochemical events, and the regulatory mechanisms including the ever-expanding number of pathways that modulate ferroptosis, such as the glutathione/glutathione peroxidase 4 (GPX4) pathway, mevalonate pathway, trans-sulfuration pathway, RAS-BRAF-MAP2K/MEK pathway, and HSF1-HSPB1-PRKC pathway [162,163,164,165].

Briefly, the first step in ferroptosis is the excessive accumulation of free iron which undergoes redox transformation between ferrous iron (Fe^2+^) and ferric iron (Fe^3+^). Fe^2+^ and hydroxyl radicals (^●^OH), the most unstable oxygen free radicals, can be directly catalyzed by the Fenton chemical reaction. Moreover, once an imbalance occurs between the ROS induced by iron enrichment and the antioxidant system, lipid peroxidation can occur, which damages the plasma membrane and induces iron death. The large concentration of Fe^2+^ that collects in the cytoplasm induces lipid hydroperoxide to generate toxic lipid ROS which, in turn, target PUFAs by catching electrons near the PUFAs, triggering a vicious reaction cycle of lipid oxidation and causing more severe oxidative damage [162,163]. Among the several pathways involved, ferroptosis can be induced by erastin, which inhibits the membrane-bound cystine/glutamate antiporter XC-. Thus, the cellular uptake of cysteine, which is an essential precursor to the synthesis of cellular antioxidant GSH, is impaired by inhibition of the antiporter. An intracellular deficit of GSH leads to ROS accumulation, membrane lipid oxidation, and subsequent cell death and elicits GPX4 inactivation, because GPX4 requires GSH as a crucial cofactor [166,167]. The importance of GPX4 in the prevention of ferroptosis has been highlighted. A study by Seiler and colleagues reported that inactivation of the inducible GPX4 gene in mice or cultured cells causes significant cell death due to excessive lipid peroxidation [168]. Most research on ferroptosis has focused on oncological diseases [169], but growing experimental evidence suggests that ferroptosis and autophagy are closely linked during other pathological states such as inflammatory injury [170], hepatic fibrosis [171], neurodegenerative diseases [172,173], and ischemia–reperfusion injury [174]. Specific types of autophagy, such as ferritinophagy, clockophagy, lipophagy, and mitophagy, promote ferroptotic cell death through degradation of the iron-storing protein ferritin, the core circadian clock protein aryl hydrocarbon receptor nuclear translocator-like (ARNTL), lipid droplets (LDs), and mitochondria, respectively [175]. The molecular mechanisms underlying autophagy-mediated ferroptosis have been extensively studied in different disease models. For example, Beclin-1, which is involved in autophagy induction and tumor suppression, was recently identified as a novel solute carrier family 7 member 11 (SLC7A11)/system XC--binding protein. Interesting studies have shown that beclin-1 inhibits SLC7A11/system XC- activity in response to erastin, facilitating ferroptosis [176] or inducing ferritinophagy [177]. Furthermore, nuclear receptor coactivator 4 (NCOA4) was identified as a cargo receptor involved in selective autophagy-dependent ferritin degradation within the mitochondria, probably because the C terminus of NCOA4 binds to a conserved surface arginine on FTH1 in phagophores [178]. NCOA4-dependent ferritinophagy promotes ferroptosis through the release of free iron from ferritin. In addition, other studies have shown that the inhibition of NCOA4 or the Atg protein inhibits ferritin degradation and free iron accumulation, thus limiting subsequent oxidative damage during ferroptosis [179,180]. In addition, the storage and degradation of LDs, complex organelles derived from the esterification of free fatty acids into triglycerides and cholesterol esters in cells, involve lypophagy and ferroptosis. The selective intracellular transport of LDs by autophagosomes for lysosomal decomposition represents an essential pathway for the regulation of cellular lipid levels and possibly results in susceptibility to cell death. Additionally, the level of LDs is negatively correlated with the occurrence of oxidative stress-induced ferroptosis [181]. Indeed, LD formation has been shown to suppress RSL3-induced ferroptosis in an in vitro model of hepatocytes. Conversely, increased RAB7A (a member of the RAS oncogene family)-dependent lipophagy promotes LD degradation, increasing the rate of lipid peroxidation-mediated ferroptosis [181]. Importantly, another contribution to the stimulation of ferroptosis is related to lysosomal cell death. Erastin can induce lysosomal membrane permeabilization and subsequent cell death through the activation of signal transducer and activator of transcription 3 (STAT-3)-mediated cathepsin B expression and release, contributing to the occurrence of ferroptosis [182]. 

Although little data on autophagy-induced ferroptosis in atherothrombosis are available, the topic is gaining particular attention. Indeed, lipid peroxidation, intraplague hemorrhage, and iron deposition are specific signs of advanced plaques and may represent indirect evidence of the involvement of the ferroptosis process. Indirect evidence that may support the possible role of ferroptosis in atherothrombosis was reported in a study conducted on apoE^−/−^ mice. The results revealed that atherosclerotic lesions in apoE^−/−^ mice overexpressing GPx4 (hGPx4Tg/ApoE^−/−^) were significantly smaller than those in control apoE^−/−^ mice. Moreover, hGPx4Tg/apoE^−/−^ mice showed a lower presence of fibrous caps and acellular areas related to reduced levels of aortic F2-isoprostane as well as reduced expression of adhesion molecules and monocytes on endothelial cells and signs of endothelial necrosis and apoptosis [183].

Recently, the role of miRNA-17-92 (miR-17e92), a multifunctional oncogenic miRNA cluster involved in tumor angiogenesis and tissue development, in the regulation of endothelial cell ferroptosis was investigated [184]. Data reported that miR-17-92 protects endothelial cells from erastin-induced ferroptosis and promotes their proliferation. Furthermore, the mechanisms by which erastin induces ferroptosis have been elucidated. Zinc lipoprotein A20, a TNF-α induced molecule, was identified as a novel regulator of endothelial cell ferroptosis. Erastin significantly upregulates zinc lipoprotein A20, inhibiting cell proliferation. The overexpression of zinc lipoprotein A20 was also able to induce ferroptosis through its direct interaction with acyl-coA synthetase long-chain family member 4 (ACSL4), a biomarker of ferroptosis that drives the sensitivity of ferroptosis by shaping the cellular lipid composition. Interestingly, the authors identified zinc lipoprotein A20 as being a direct target of miR-17-92 and reported that miR-17-92 overexpression is capable of inhibiting zinc lipoprotein A20 in endothelial cells [184]. In the work of Bai and colleagues, the presence of ferroptosis and its potential effects were investigated in ApoE^−/−^ mice with high fat diet induced atherosclerosis. The results showed that the administration of ferrostatin-1, a ferroptosis inhibitor, reduced the development of atherosclerotic lesions, inhibited iron accumulation and lipid peroxidation, and inverted the expression of the ferroptosis indicators SLC7A11 and GPX4. Furthermore, ferrostatin-1 administration was shown to significantly increase cell viability in oxLDL-treated mouse aortic endothelial cells, decreasing the iron content and rate of lipid peroxidation and upregulating the SLC7A11 and GPX4 levels. Additionally, ferrostatin-1 was shown to downregulate the expression of adhesion molecules and upregulate the expression of eNOS [185].

## 7. Conclusions

As partially reported, experimental evidence from in vitro and in vivo models clearly shows the major role of autophagy in modulating the endothelium during atherogenesis and atherosclerotic plaque development and stabilization. Furthermore, the autophagic process is multifaceted, strictly dependent on the oxidative state, and differentially modulated according to the cell type. 

The stimulation of basal autophagy has been widely reported to exert clear atheroprotective actions during the early stages of atherosclerosis development within the endothelium, protecting cells against oxidative stress by degrading damaged intracellular material. Insufficient autophagy or hyperactivated autophagy in the advanced stages of disease causes plaque destabilization, leading to endothelial cell injury and death and promoting the development of lesional thrombosis [131]. Interesting studies have demonstrated that oxLDL production and accumulation occurring at the site of endothelial damage inhibit mTOR activity, thus upregulating the protein levels of beclin-1 and LC3II, resulting in autophagosome formation and subsequent engulfment and degradation of excess lipids [135,137]. On the contrary, the inhibition of endothelial autophagy through several genetically engineered experimental models of conditional Atg deletions confirmed the exacerbation of the atherosclerotic process [136,141,143,150]. 

However, several studies have reported that excessive oxLDL production along with the upregulation of LOX-1 expression can lead to defective autophagic flux, characterized by either beclin-1 and LC3II inhibition or by hyperactivation of these markers and triggering inflammatory and oxidative stress responses [147,148]. 

Alongside the increases in beclin-1 and LC3II, several studies have shown an increase in the protein level of p62 within the endothelium following oxLDL overexpression, suggesting that the upregulation of p62, which is selectively degraded during physiological autophagic flux, results in impaired autophagic degradation [186,187].

Defective autophagy flux can contribute to the development of atherosclerotic plaques, promoting disturbed endothelial alignment in response to flow, apoptosis, and a senescent phenotype in ECs [142,143,144,145,146]. Additionally, in vivo experiments have shown that, in the plaque endothelium, the activation of mTOR leads to the inhibition of autophagy, protecting ECs and stabilizing atherosclerotic lesions [141]. 

Likewise, in advanced atherosclerotic plagues, iNOS upregulation, the aging process, or ceroid deposition can impair efferocytosis, resulting in increased sensitivity to apoptotic stimuli, crystal formation, and inflammasome hyperactivation [132,133,134,135,136,137,138].

Moreover, recent studies interestingly identified some non-coding RNAs that are differently involved in the modulation of the autophagic pathway within ECs during atherosclerotic development. These reports demonstrated that non-coding RNAs represent an important connection between autophagy and endothelial dysfunction [152,153,154,155]. An important topic that is gaining particular attention concerns autophagy-induced ferroptosis in atherothrombosis. Most of the research in this area has focused on oncological diseases, but a growing body of experimental evidence suggests that there are also relationships between ferroptosis and autophagy in inflammatory injury [170], hepatic fibrosis [171], neurodegenerative diseases [172,173], and ischemia–reperfusion injury [174]. Although data related to the role of autophagy-induced ferroptosis in the onset of atherosclerosis are still limited, some characteristic features of the atherothrombotic process, such as lipid peroxidation, intraplaque hemorrhage, and iron deposition, may represent indirect evidence of the involvement of the ferroptosis process [183,184,185].

Despite the growing body of knowledge collected in recent years, further in-depth studies are necessary to better clarify the importance of this phenomenon in human atherosclerotic plaques and to recognize the critical regulatory molecular mechanisms underlying it.

On this basis, it will be possible to identify certain molecular targets and thus translate these notions into the clinical field to form future preventive and therapeutic interventions.
